# Friction and Forces in Orthodontics: Understanding Space Closure

**DOI:** 10.7759/cureus.65270

**Published:** 2024-07-24

**Authors:** Nikita Soni, Priyanka Niranjane, Ranjit Kamble, Akanksha Purohit

**Affiliations:** 1 Department of Orthodontics, Sharad Pawar Dental College and Hospital, Datta Meghe Institute of Higher Education and Research, Wardha, IND; 2 Neglected Tropical Diseases, Global Health Strategies, Delhi, IND

**Keywords:** friction mechanics, frictionless mechanics, opus loop, loop mechanics, space closure

## Abstract

Orthodontic space closure is a critical aspect of treatment aimed at the correct positioning of teeth and is linked to tooth movement and optimal biomechanics. Therefore, the goal of this case study is to elucidate the process, describing the challenges encountered and the solutions adopted, with a focus on the frictionless technique and the use of devices like the Opus Loop to close spaces. Sliding mechanics, known for high friction, and segmental mechanics, characterized by low friction and continuous adjustment, are two significant technologies used. In this specific case, the frictionless methods applied to a 23-year-old female patient with protruding superior labial incisors included: extraction of the first premolars in all four quadrants, followed by consecutive wiring. Retraction was performed using an Opus Loop, significantly improving the patient's facial profile and dental arch over the next year and a half. As a result, the study demonstrates that the Opus Loop greatly reduces friction forces and offers an effective mechanism to influence tooth movement in orthodontic treatment regimens.

## Introduction

Orthodontics aims to straighten teeth through space closure. This arduous process involves meticulously shifting teeth to close spaces and realign them. However, achieving optimal space closure can be challenging. Orthodontists often rely on their understanding of biomechanics to achieve the desired results and ensure proper tooth movement [1]. In this case study, we examine loop mechanisms used for space closure: sliding mechanics and segmental/sectional mechanics. Friction plays a significant role in orthodontics, referring to the force that prevents motion between two surfaces [2]. Friction during space closure occurs because the archwire used in orthodontics comes into contact with either the bracket slot or ligature [3]. High frictional forces can hinder tooth movements, thereby causing delays in treatment because it takes longer for teeth to move. Therefore, it is imperative to understand and control friction during treatment, especially during space closure [4].

Orthodontic tooth movement is governed by two main mechanisms: sliding mechanics, also known as friction mechanics, involve multiple teeth being pushed together straightly with a wire of the same size for each bracket [5]. Although sliding mechanics is straightforward, the occurrence of friction between the wire and bracket surfaces is inevitable [4]. Segmental mechanics, popularly referred to as frictionless mechanics, use selective forces to move single segments of teeth. This technique involves bending specific parts of the archwire, which reduces friction and affords greater control over individual tooth movements. Different factors such as the complexity of the case, the need for reduced treatment time, and the type of desired tooth movement pattern influence the choice between these techniques. This case study will discuss the pros and cons of each method to achieve desirable space closure, considering issues like friction control, treatment efficiency, and potential side effects [4].

## Case presentation

**Figure 1:**
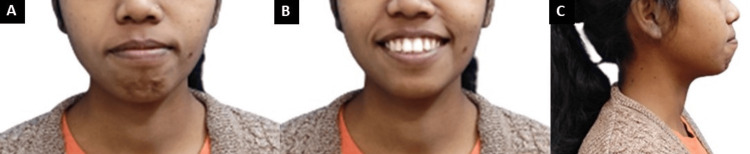
Extra-oral photographs: (A) frontal, (B) smiling, (C) profile.

A patient, a 23-old female, visited the orthodontic department nineteen months ago with a complaint about her protruding upper anterior teeth. The pre-treatment extraoral examination revealed a mesoprosopic face form and a mesocephalic head form. Her profile exhibited a convex shape, with lips that were more protuberant on the bottom than on top, and an upper lip that appeared shorter when viewed from the side. The nasolabial angle of this individual was average (normal), her lips were competent, and her mentolabial sulcus was shallow (Figure 1).

**Figure 2:**
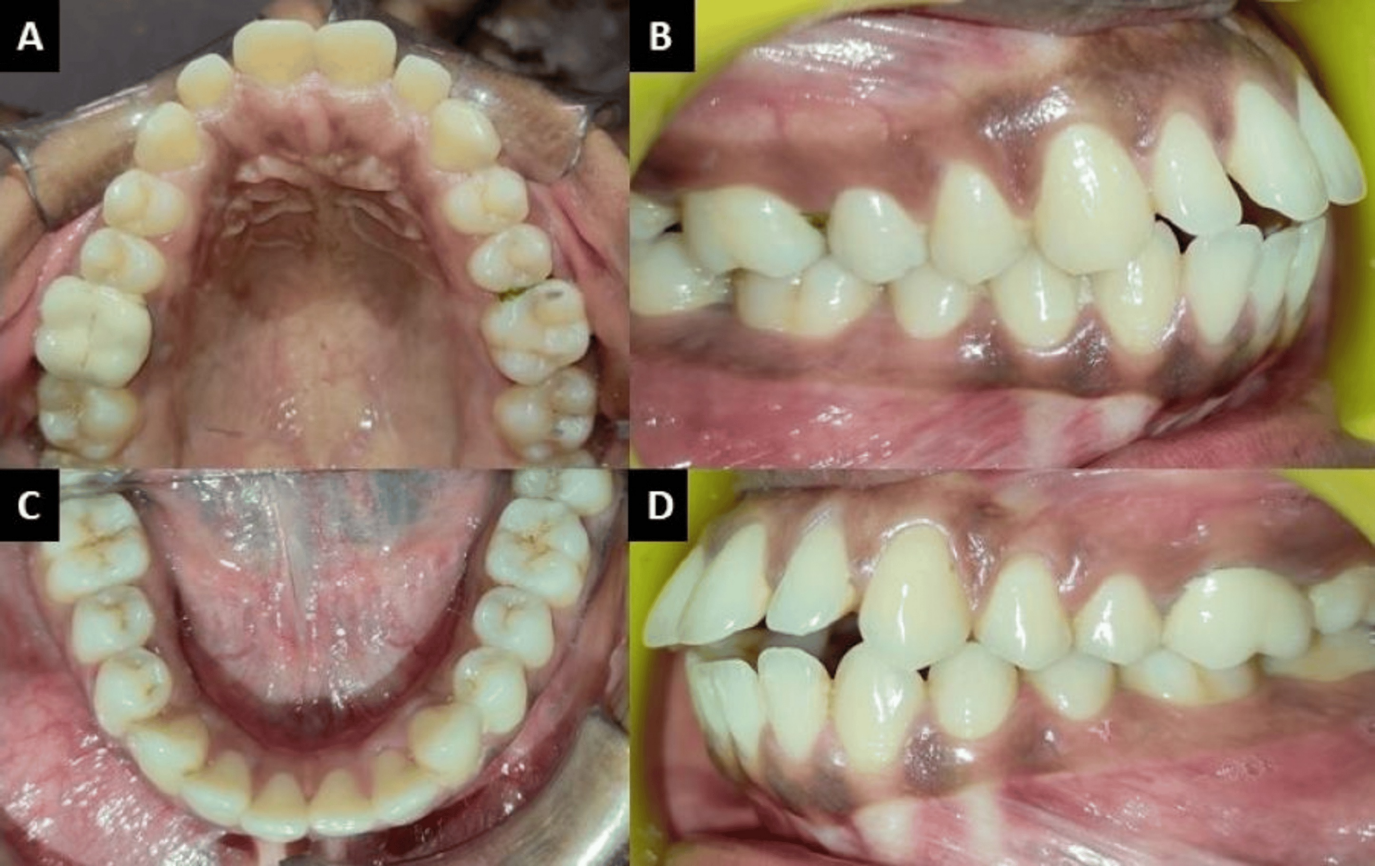
Intra-oral photographs: (A) maxillary arch, (B) right lateral, (C) mandibular arch, (D) left lateral.

The intraoral examination showed that all permanent teeth, including third molars, had erupted. The upper and lower anterior teeth were proclined. Both the upper and lower arches were U-shaped with Class I molar and canine relationships on both the right and left sides. The lower midline was shifted to the left, and an anteriorly open bite of 2-3 mm was observed (Figure 2).

**Figure 3:**
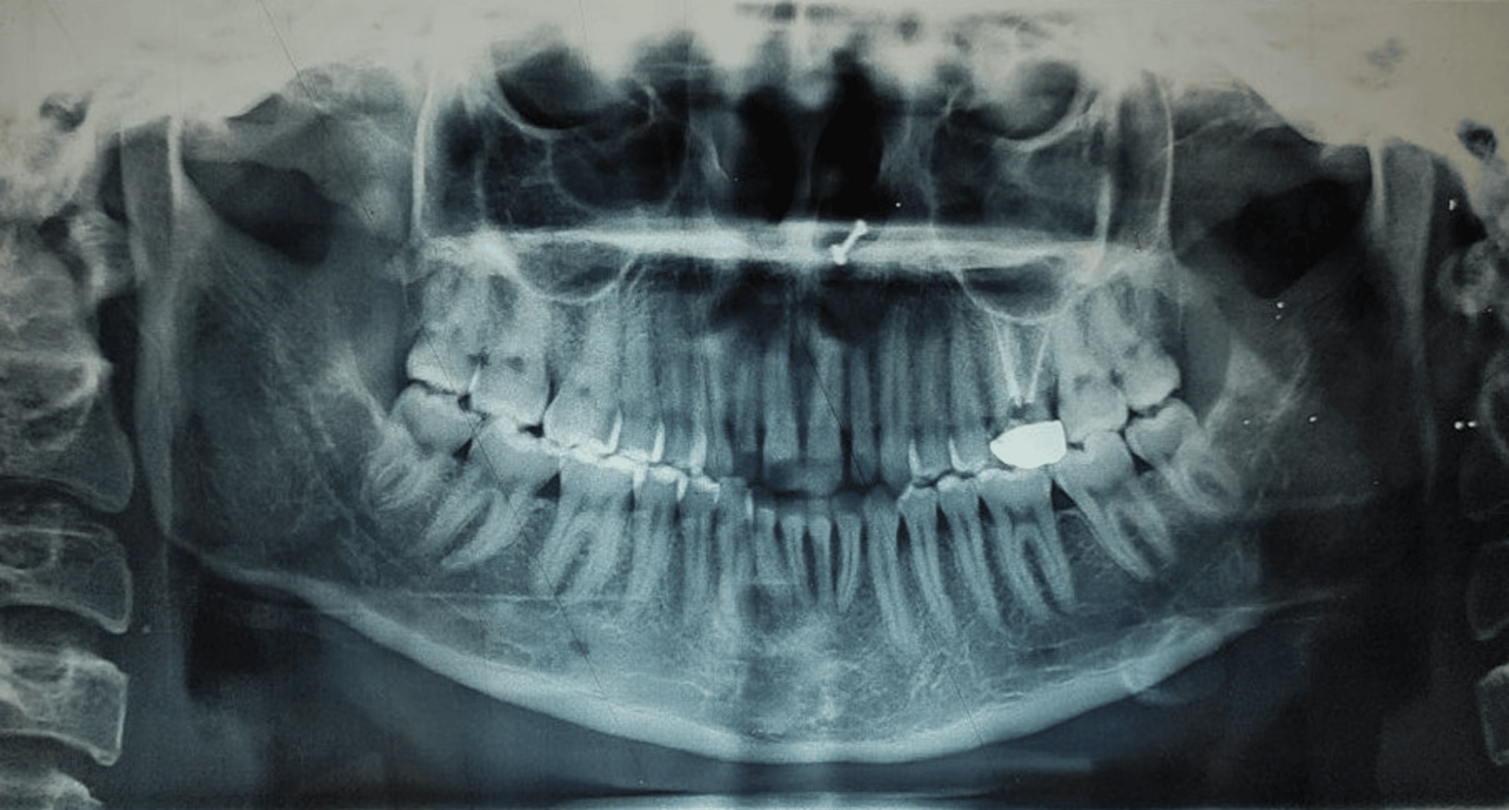
Pretreatment orthopantomogram.

The orthopantomogram (OPG) before treatment showed that all teeth in all four quadrants were present, including the third molars. The first molar in the second quadrant had undergone root canal treatment six months prior. Temporomandibular joint (TMJ) on both sides appeared normal with good bone health around the teeth (Figure 3).

**Figure 4:**
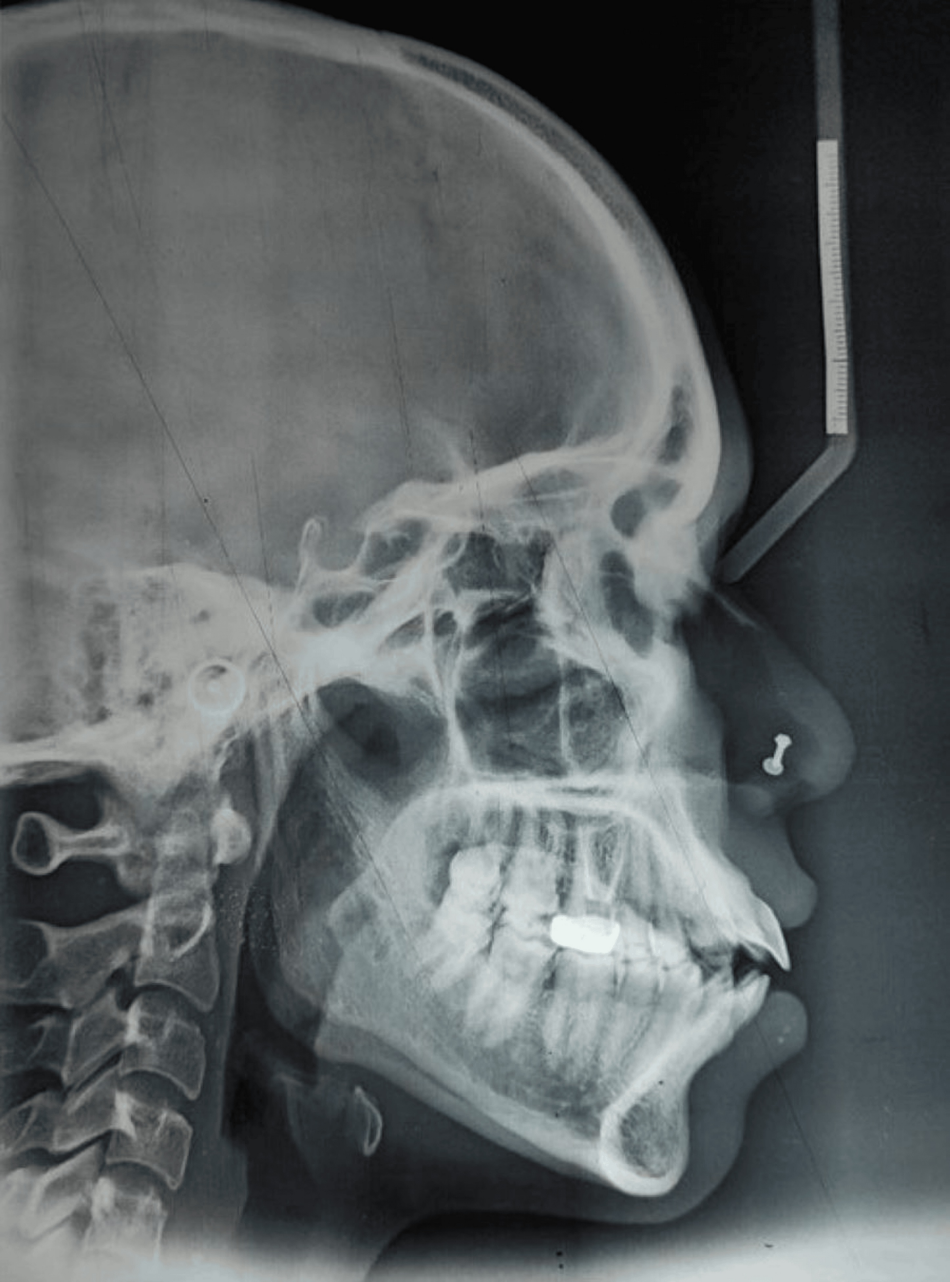
Pretreatment lateral cephalogram.

Cephalometric analysis indicated that the patient was in cervical vertebral maturational indicator (CVMI) stage VI (maturation) and had class I skeletal bases with a horizontal growth pattern. There was an increased proclination of the upper incisors by 14° and 8 mm, and of the lower incisors by 17° and 7 mm. Soft tissue analysis revealed a normal nasolabial angle and protrusive upper and lower lips (Figure 4).

Model analysis interpreted that there was 12 mm of proclination in the maxillary arch and 8 mm in the mandibular arch. Bolton’s ratio revealed a mandibular excess with an anterior ratio of 82.60% and an overall ratio of 92.63%. There was a space discrepancy of 12 mm in the maxillary arch and 10 mm in the mandibular arch.

The treatment objectives were to correct the anterior open bite, reduce the proclination of the incisors in both arches, correct the midline shift, achieve a normal overjet and overbite, and improve the profile. The treatment plan to achieve these objectives involved retracting the maxillary and mandibular incisors following the extraction of all first premolars in both arches.

Orthodontic treatment

**Figure 5:**
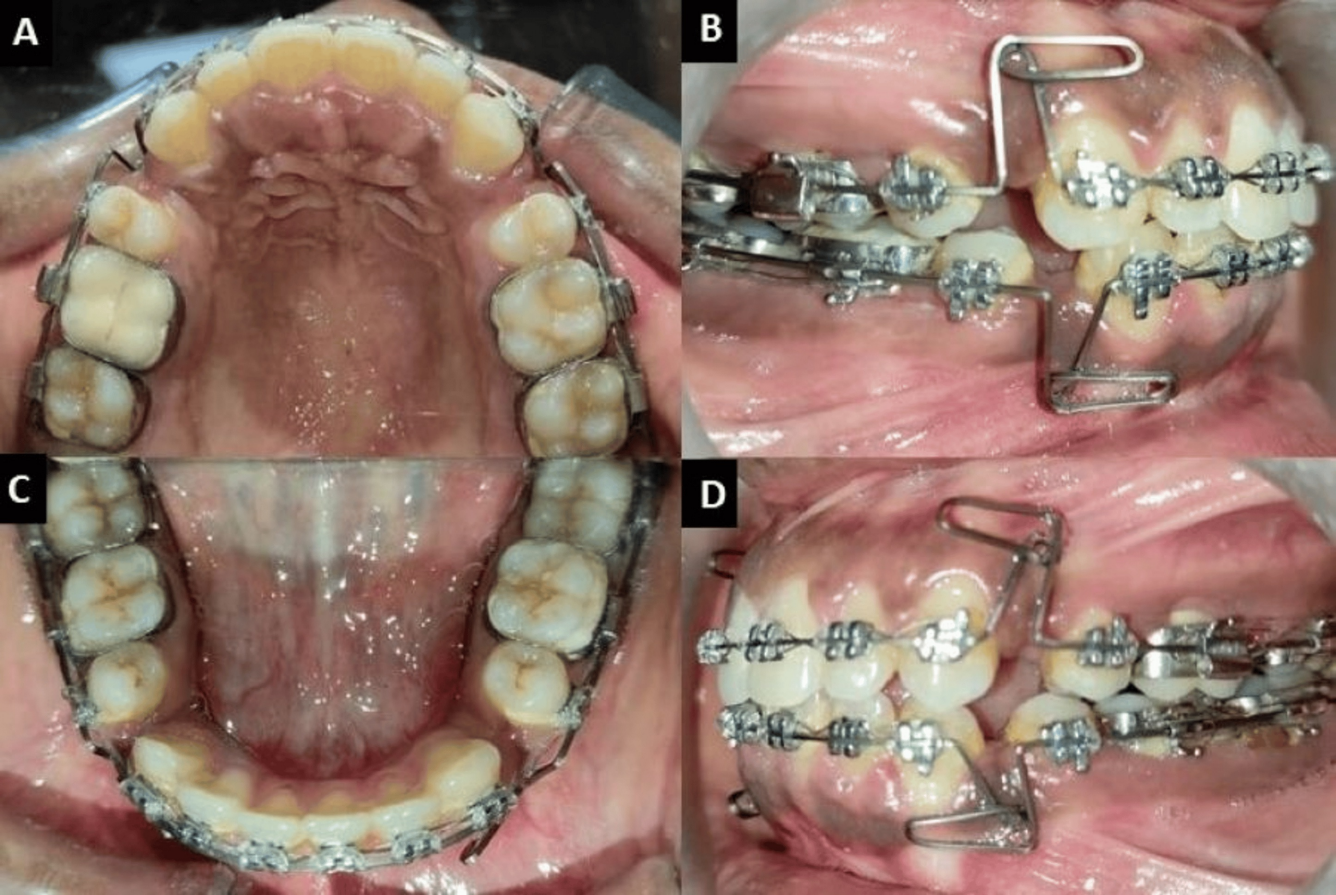
(A and C) Occlusal view and (B and D) in-occlusion ligated Opus loop.

All teeth were bonded and banded using an Mclaughlin Bennet and Trevisi (MBT) 0.022 slot prescription in the upper and lower arches. Extractions of all first premolars in all quadrants were performed. Initial leveling and alignment of the arches began with sequential wiring, starting from round wires followed by rectangular wires, and was completed within 4-5 months. After this, the anterior tooth segments of the maxilla and mandible were retracted using a continuous 0.017×0.025-inch titanium molybdenum alloy (TMA) Opus loop. The loop was activated extraorally, ligated to each bracket slot, and placed on molar tubes (Figure 5). After seven months of retraction of the anterior segments in the upper and lower arch using the Opus loop, the extraction space was closed.

Results

**Figure 6:**
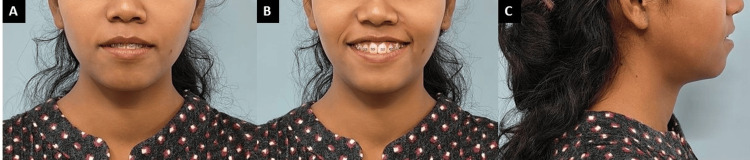
Post-treatment extraoral photographs: (A) frontal, (B) smiling, (C) profile.

**Figure 7:**
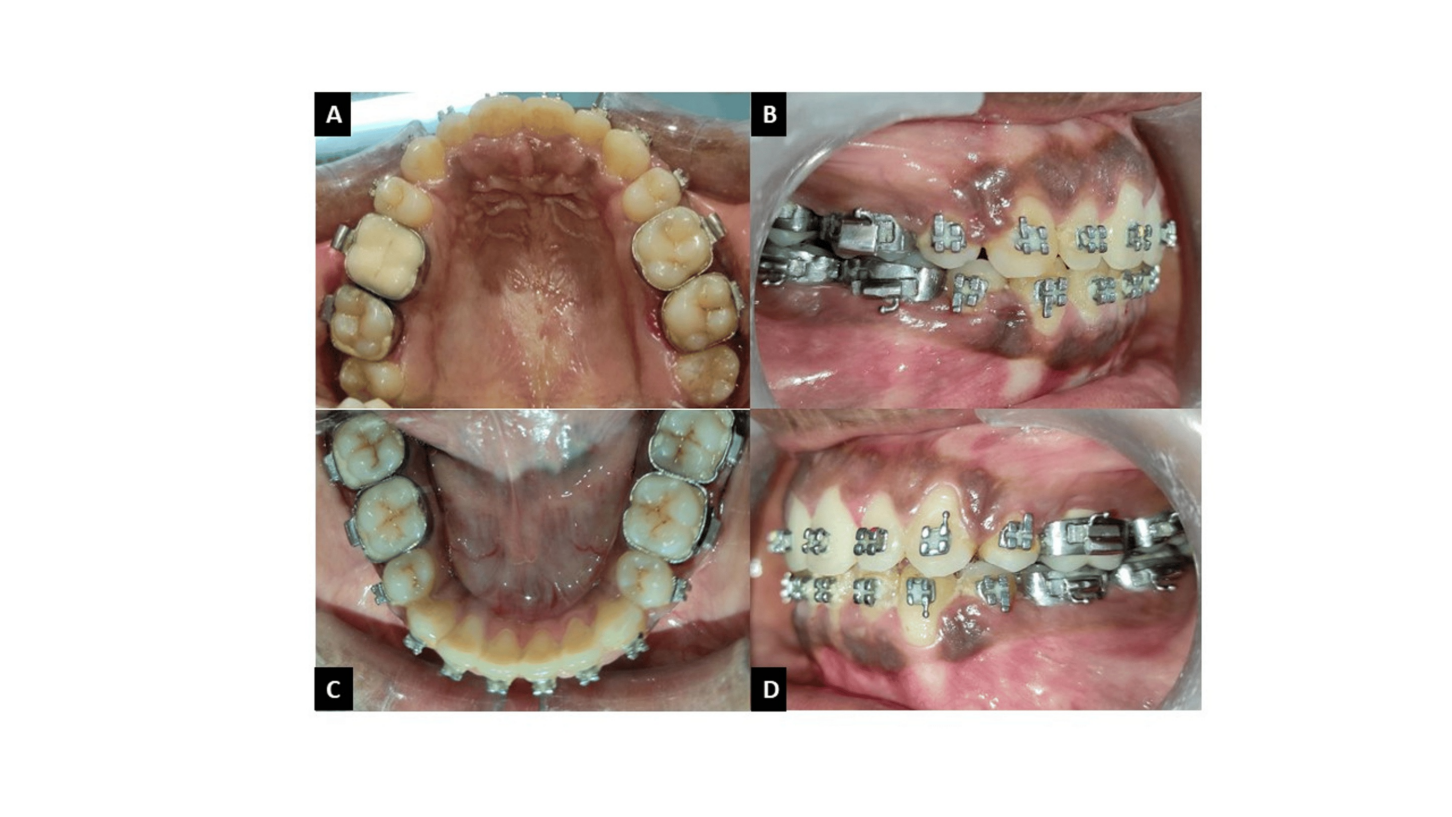
(A) Maxillary arch, (B) Right occlusion, (C) Mandibular arch, (D) Left occlusion.

**Figure 8:**
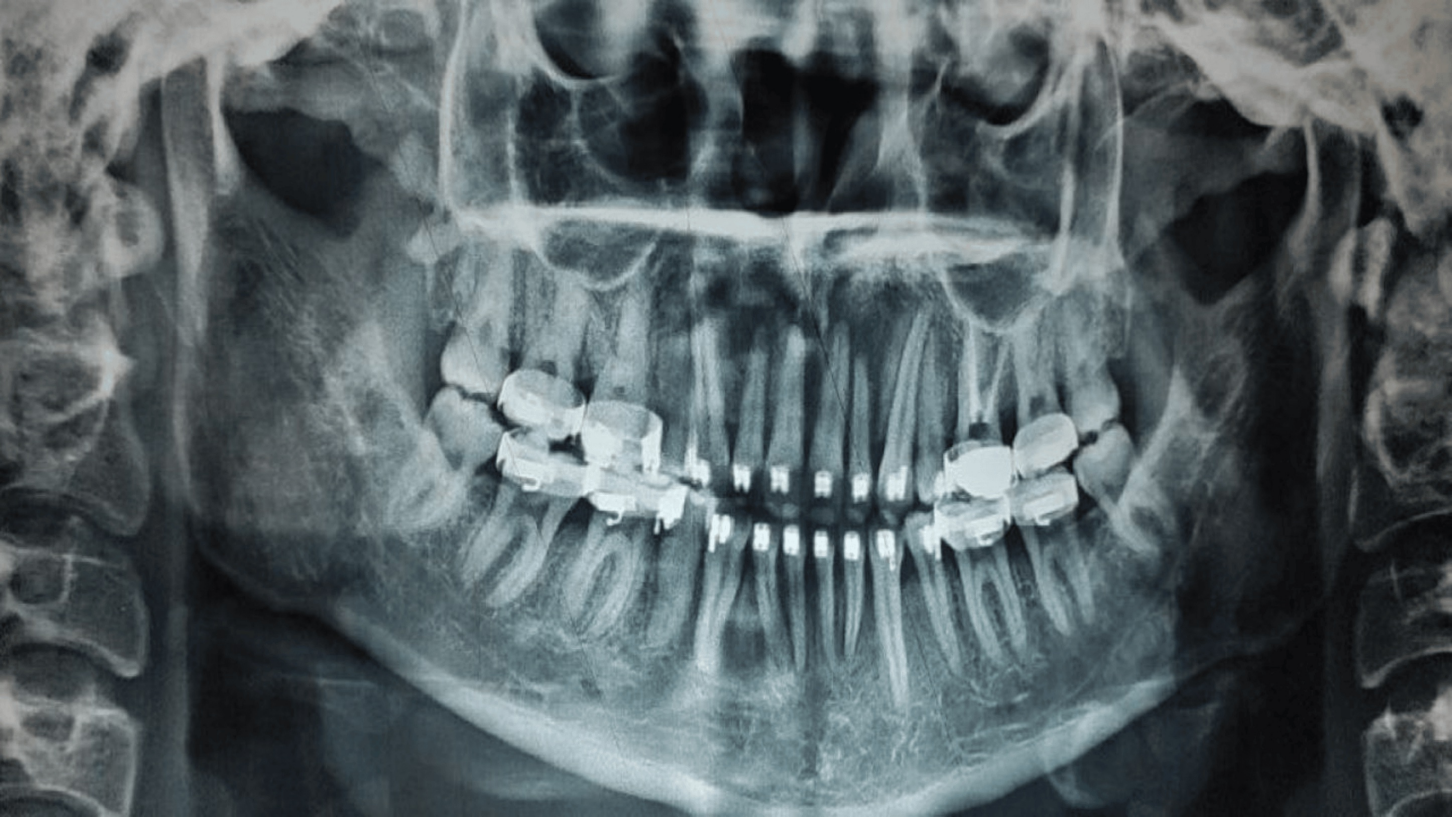
Post-treatment orthopantomogram.

**Figure 9:**
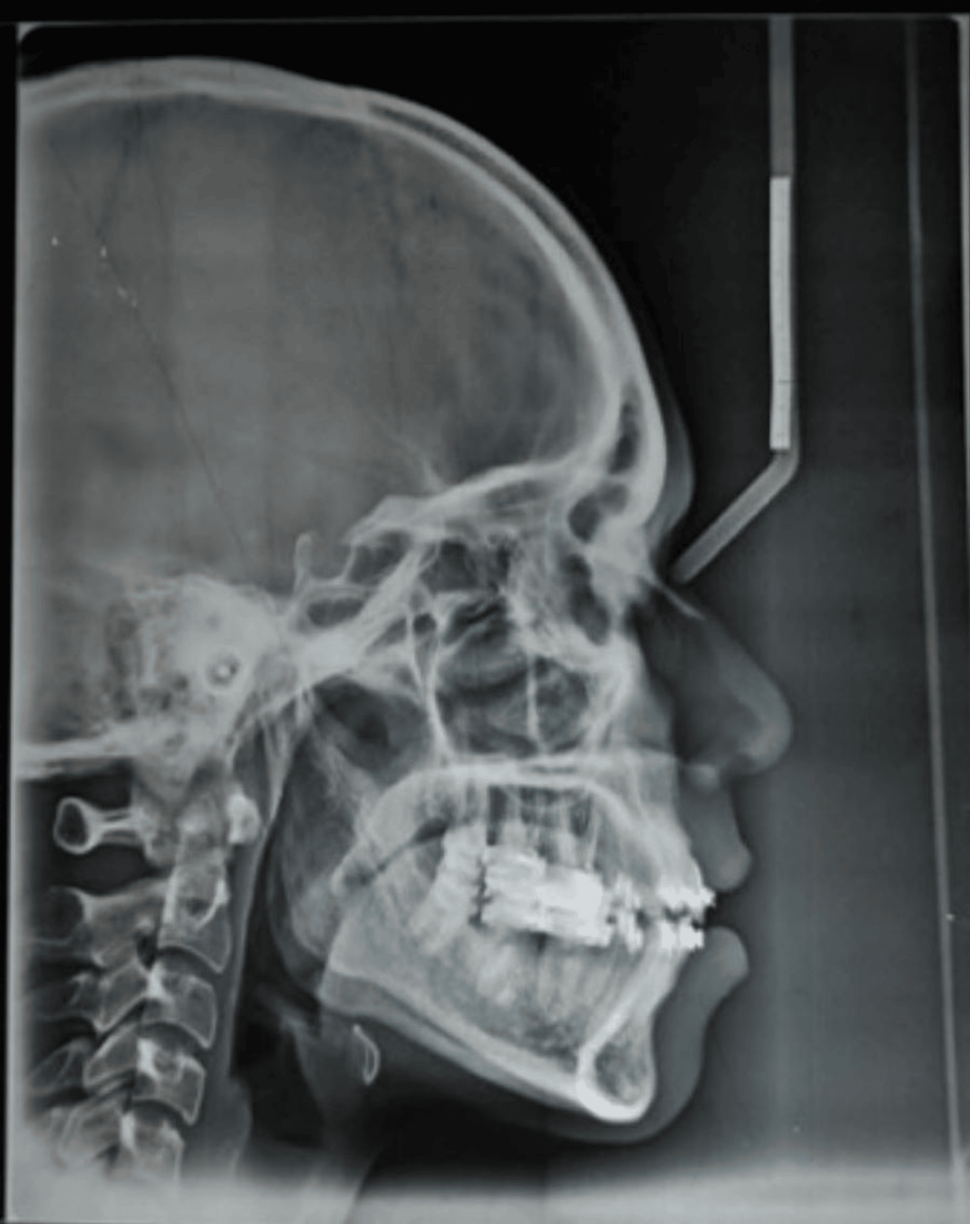
Post-treatment lateral cephalogram.

After 18 months of conventional orthodontic treatment, the post-treatment records showed marked improvement in the facial profile and the patient’s smile (Figure 6). Protrusion of the maxillary and mandibular anterior teeth was corrected using the Opus loop while maintaining a Class I molar and canine relationship. The anterior open bite was corrected while maintaining normal overjet and overbite. The angles between SN-upper incisors and SN-lower incisors were significantly decreased, contributing to the correction of the open bite and the convex profile (Figures 7-9).

## Discussion

In this complex orthodontic scenario, a meticulous and methodical strategy was crucial for optimizing extraction space closure. Acknowledging the variability in the optimal force for tooth movement, which ranges from 75 to 260 grams as noted by Gajda S and Chen J, a consistent force of approximately 130 grams was targeted for retracting the maxillary anterior teeth [6]. Traditional friction mechanics, involving the sliding of teeth along an archwire, were considered suboptimal due to their higher force requirements and the inconsistent force delivery stemming from frictional resistance [7]. Consequently, frictionless mechanics utilizing retraction loops were selected for their superior control over tooth movement and stable force application, despite necessitating precise mechanical skills and additional chair time.

Regarding the frictionless mechanics, the Opus loop was selected as it falls into this system category, offering precision and control in the mechanism of highly effective extraction space closure. Theoretical, conceptual, and simulation computer analysis of the Opus loop indicated that it was mechanically superior, especially regarding loop height for an enhanced angle of gradient length. Moment to Force ratio analysis using the Castigliano theorem for Opus loops is higher than that for conventional vertical loops, as confirmed by Finite Element Method (FEM) [8]. These results suggest that there may be an optimality when it comes to the size of loops and the angles of tendrils. It is reported that the larger loop height and length combined with the smaller radius results in the maximum efficiency of the Opus loop’s action [9], making this type of loop ideal for orthodontic space closure due to the highest refinement of its mechanical properties.

When selecting loops for an extraction space closure, the force and moment coefficients applied to the left maxillary lateral incisor and canine were compared. The designs of the Opus loops demonstrated a high ability to reduce unfavorable tooth shifts compared to other loop designs. Due to the closing and opening forces and moments created by Opus loops, the displacement of teeth is more manageable and predictable with less risk of intrusive and extrusive forces, thereby reducing the occurrence of unfavorable side effects during treatment. In summary, Opus loops provided better stabilizing properties and are less prone to undesirable rotational and tipping motions.

Furthermore, Opus loops possessed higher moment-to-force ratios, especially in matters of the bodily movement of teeth, with maximum values of 8.7 [9]. The load/deflection ratios varied from 1103 ± 173 near the premolar bracket to 1797 ± 222 near the canine bracket for Opus loops. Greater force control was observed with Opus loops, which produced less variation in vertical forces than T-loops, thus providing enhanced mechanical efficiency for extraction space closure in orthodontic treatments [9].

Evaluation of the changes in dental and soft tissues during treatment with Opus loop mechanics and extraction space closure, compared to temporary anchorage devices (TADs), demonstrated that both techniques significantly impacted dental and soft tissues from the beginning to the end of the treatment, with negligible changes to the skeletal framework. However, the Opus loop group exhibited greater retraction of the lower anterior teeth and better profile esthetic changes than the TAD group. Regarding horizontal measurements, it was revealed that loop mechanics allowed for more bodily movements of the teeth, and subjects tended to tip their upper incisors less with their tongues compared to those treated with sliding mechanics. Vertical analysis showed that the upper incisors in the loop group were more intruded and the occlusal plane was flatter, while the sliding group displayed slightly more clockwise rotation and less intrusion. These findings suggest that the advantages of loop mechanics in terms of precise control of tooth movement and stability of the occlusal plane are superior to those of TADs in orthodontic treatments [10].

## Conclusions

This particular case report highlights the effectiveness of frictionless mechanics, specifically the Opus loop, in orthodontic space closure following the extraction of the first premolars. The application of the Opus loop to the attachments provided excellent control of the force system needed for tooth movement, minimizing unwanted rotational and tipping forces and yielding more predictable and faster treatment outcomes. This approach significantly alleviated the patient’s initial concerns about upper front teeth protrusion and anterior open bite, ultimately exerting a profound positive influence on the latest assessments of the patient’s profile and dentition. In comparison with traditional sliding mechanics and TADs, the Opus loop also demonstrated superior accuracy and efficiency in tooth retraction with fewer side effects. These results will support the continued practice and development of frictionless mechanics in orthodontic practice as a stable and effective tool for space closure and other successful treatment outcomes.
